# *Helicobacter pylori* outer membrane vesicles involvement in the infection development and *Helicobacter pylori*-related diseases

**DOI:** 10.1186/s12929-018-0480-y

**Published:** 2018-11-08

**Authors:** Magdalena Chmiela, Natalia Walczak, Karolina Rudnicka

**Affiliations:** Laboratory of Gastroimmunology, Department of Immunology and Infectious Biology, Faculty of Biology and Environmental Protection, University of Łódź, Banacha 12/16, 90-237 Łódź, Poland

**Keywords:** *Helicobacter pylori*, outer membrane vesicles, virulence factor, inflammation, gastritis

## Abstract

*Helicobacter pylori* - (*H. pylori*) play a role in the pathogenesis of gastritis, gastric and duodenal ulcers as well as gastric cancer. A possible involvement of outer membrane vesicles (OMVs) produced by *H. pylori* in the distribution of bacterial antigens through the gastric epithelial barrier and their role in the development of local and systemic host inflammatory and immune responses has been suggested. OMVs contain various biologically active compounds, which internalize into host cells affecting signaling pathways and promoting apoptosis of gastric epithelial and immunocompetent cells. OMVs-associated *H. pylori* virulence factors may strengthen or downregulate the immune responses leading to disease development. This review describes the biological importance of *H. pylori* OMVs and their role in the course of *H. pylori* infections, as well as *H. pylori* related local and systemic effects.

## Introduction

### The pathogenicity of *Helicobacter pylori*

*Helicobacter pylori* (*H. pylori*) are Gram-negative microaerophilic, spiral-shaped bacteria that belong to the ɛ subdivision of the *Proteobacteria*, order *Campylobacterales*, family *Helicobacteriaceae.* Due to ability to adhere to gastric epithelium or internalize inside the cells they colonize human gastric mucosa, and cause gastritis, gastric ulcers and even cancers, or even recently considered development of extragastric diseases [1–5]. The pathogenicity of *H. pylori* and the ability to cause various diseases depend on the high genomic variability of these bacteria [6].

The prevalence of *H. pylori* infections is associated with a geographical region or living conditions. Moreover the demographic flux of human populations induce the occurrence of new *H. pylori* genetic variants [7–14].

These bacteria are well adapted to the harsh environment of the gastric niche due to motility, urease production, variability of adhesins, different effector molecules, and the Lewis (Le) blood group antigens (Le^x^ and Le^y^) expression, which determines molecular mimicry, and immune evasion [2, 3, 6, 15, 16]. During the acute phase of colonization *H. pylori* resist the oxidative stress caused by excessive inflammatory response and in the chronic phase of infection, these bacteria downregulate the host immune responses driving the immunity towards tolerance rather than to a protective response [17–22]. Recently, it was shown that *H. pylori* replicate in murine bone marrow derived dendritic cells and on this way can affect immune responses mediated by these population of antigen presenting cells [23].

*H. pylori* virulence factors include cell-bound components, externally secreted molecules and antigens that need to be introduced to a host cell by the type IV secretion system (T4SS) such as cytotoxin-associated gene A (CagA) protein [20, 24–26]. However, similarly to other Gram-negative bacteria, *H. pylori* also produce outer membrane vesicles (OMVs) that contain various virulence factors and this strategy supports the survival of these bacteria in the gastric mucosa [27, 28]. The studies on specific *H. pylori* compounds delivered by OMVs and on their role in the pathogenicity of these bacteria are ongoing.

## General aspects of bacterial OMVs

### Mechanism and conditions of bacterial vesiculation

Outer membrane vesicles are defined as 20–300 nm blebs, which are naturally secreted by Gram-negative bacteria mainly during their logarithmic phase of growth although, Gram-positive bacteria also form the extracellular particles [29–34]. The models of OMVs biogenesis include: accumulation of peptidoglycan particles in the periplasm, which causes a turgor pressure; arising vesiculation in the relaxed cell wall-outer membrane regions as well as anionic repulsion and destabilization of outer membrane [35–38]. Recently, a novel mechanism of vesiculation has been proposed. It involves a VacJ/Yrb ABC (ATP-binding cassette) system - a potential phospholipid transporter engaged in phospholipid accumulation in the outer membrane [39]. OMVs produced by individual bacteria as well as those remaining in biofilms contain mainly periplasmic proteins, outer membrane proteins (OMPs) as well as lipids. In addition, some OMPs carry an extracellular DNA (eDNA), which plays a role in their aggregation during biofilm formation [32, 40]. Some bacterial components do not remain within OMVs and others like heat-labile enterotoxins of enterotoxigenic *Escherichia coli* (ETEC) and aminopeptidase of *Pseudomonas aeruginosa* are elevated in vesicles, when compared to their expression in whole bacteria [35]. The genes specific for all strains that are responsible for the maintenance of the cell membrane organization and vesicle formation are localized within the *tol-pal* cluster [41].

The process of vesiculation is influenced by stress conditions such as increased temperature or the lack of nutrients. This phenomenon is genetically regulated and determines the interaction of the pathogen with host tissues and proteolytic enzymes, which was proved by showing the presence of bleb-like evaginations on the surface of *Pseudomonas fragi* observed within the damaged muscles of pigs exposed to these bacteria [42–44].

### The immunomodulatory potential of bacterial OMVs

Previously, it was thought that OMVs promote survival of nonpathogenic species, while in pathogenic species enhance their virulence, the ability to downregulate or stimulate host immune defense, and raise the pathogenic potential [30, 40, 45–47]. However, recent studies showed that microbiota-derived OMVs carry various compounds, which can influence diverse signaling pathways in the host cells [48, 49]. This can explain the role of human microbiota in the development of many diseases such as inflammatory bowel disease, metabolic disorders including diabetes, obesity, liver disease as well as autoimmune and cardiovascular or allergic diseases.

Proteomic analysis of Gram-negative bacteria showed that bacterial structures present in OMVs play a role in intercellular bacteria-bacteria and bacteria-host interactions, whereas secreted OMVs are capable of interacting with and adhering to host cells [50]. They constitute the antigen delivery system alternative to well known bacterial secretion systems [27, 45]. OMVs, which resemble the bacterial surface compounds, can serve as decoys for antibodies and defensins produced in the host, which can unable the bacterial persistence [51, 52]. They are involved in bacterial survival, DNA transfer, acquisition of antibiotic resistance and induction of immune and non-immune cell apoptosis [47, 53–56]. Clinical studies showed anti- and pro-inflammatory properties of OMVs. The polysaccharidic components of *Bacteroides fragilis* OMVs exhibited anti-inflammatory properties in patients with colitis due to upregulation of regulatory T cells. On the contrary, pro-inflammatory potential of *Moraxella catarrhalis* OMVs was shown in patients with chronic obstructive pulmonary disease as well as meningococcal OMVs in sepsis [57–59]. Since bacterial outer membrane compounds display adjuvant-like effects the attempts were made to use OMVs in vaccination protocols [45, 60–63].

## *H. pylori* outer membrane vesicles

### Major components of *H. pylori* OMVs

*H. pylori* OMVs crossing the cellular barrier are considered as the effective way of *H. pylori* antigens translocation through the gastric mucosa to immune cells in tissues or even to circulation. This mechanism of antigen transport seem to be an alternative for the TSS4 secretion system or to direct secretion of soluble compounds to the surrounding environment [4, 23, 25, 64].

Olofsson *et al*., using *H. pylori* OMVs separated by density gradient centrifugation and probed for inner membrane (IM) and outer membrane (OM) markers demonstrated that vesicles of these bacteria contain OM, but not IM compounds [27]. Blebbing of *H. pylori* OM was detected by electron microscopy both in gastric biopsies of infected individuals and in cultures of these bacteria especially during the late stationary growth phase, and during biofilm formation [40, 46, 65].

The content of OMVs might vary among particular *H. pylori* strains as they are encoded by various genes. In *H. pylori* OMVs the following phospholipids were found: phosphatidylglycerol (PG), phosphatidylethanolamine (PE), lyso PE (LPE), phosphatidylcholine (PC), lyso PC (LPC) and cardiolipin [27]. Lipopolysaccharide (LPS) anchored in the outer membrane by lipid A is another important component of *H. pylori* OMVs. The presence of Lewis^XY^ antigens in an O-specific region of *H. pylori* LPS depends on iron-reach conditions, whereas the presence of Lewis^Y^ determinants on iron-limited conditions [66–69].

Proteins of *H. pylori* OMVs are divided into five families, containing Hop, Hor, Hof, Hom proteins as well as iron-regulated OMPs and efflux pump OMPs [70]. The following proteins have been identified: vacuolating cytotoxin (VacA), CagA, blood group antigen binding adhesin (BabA), sialic acid binding adhesin (SabA), outer inflammatory protein A (OipA), *H. pylori* neutrophil activating protein (HP-NAP), adherence associated lipoprotein (AlpA), and urease [27, 71]. Most of *H. pylori* OMVs proteins play a role in adherence, corresponding with the elevated gastric epithelial cell damage and possibly with the gastric cancer development [72–81]. The prevalence of these virulence factors differs among *H. pylori* isolates from various geographic areas, which may be reflected in the incidence of gastric cancer. There is a large intercountry variation in incidence of gastric cancer and *H. pylori* seroprevalence among Asian countries. In Japan, and Taiwan, the infection rate is 39.3%. By contrast, the prevalence of *H. pylori* infection is high in Vietnam (74.6%) and Bangladesh (92%), but in these regions low gastric cancer rates have been reported. This has been termed the ‘Asian enigma’. Interestingly the majority of *H. pylori* strains isolated from Asian population are *babA2* gene positive [82–84].

Fujimoto and colleagues showed that *H. pylori* infection with BabA-L strains was associated with highest risk of gastric cancer, followed by BabA-H and BabA-negative strains. In Western countries, infection with BabA-L and BabA-H strains are associated with 54.8-fold and 19.8-fold risk of gastric cancer compared to BabA-negative infectors. Moreover, infection with *H. pylori* BabA-L-positive strain is related with higher colonization rate, neutrophil infiltration, and mucosal atrophy. The involvement of these OipA proteins is not so clear since all *H. pylori* isolates from East Asia are either BabA-H or BabA-L [85]. Furthermore, it was showed that although *babA2*-genonegative strain was associated with lowest gastric cancer risk in *babA2*-genopositive strain, a lower BabA expression level seemed to be associated with higher gastric cancer risk [85, 86]. In East Asia a high risk *H. pylori* markers for gastric cancer development include CagL Y58E59 vs non-Y58E59, vacA *s1*, vacA *i1*, *m1* and EPIYA-D motif of CagA [87–89].

Apart from these virulence factors (released to the gastric milieu or transported via OMVs) several host-dependent determinants were shown to be involved in higher incidence of gastric cancer. There is a strong association between genetic polymorphisms in the interleukin (IL-)1β and IL-1 receptor gene cluster and in tumor necrosis factor (TNF)-α and the risk of gastric cancer [90, 91]. Interestingly a meta-analysis of the role of IL-1β and it's antagonist gene polymorphisms in gastric cancer risk showed an association in Caucasians, but not in Asian populations [92]. In the context of gastric carcinogenesis, apart from OMVs, bacterial virulence factors that have been implicated include CagA and VacA. Meta-analysis of the relationship between CagA seropositivity and gastric cancer performed by Huang and colleagues showed that infection with CagA-positive strains of *H. pylori* increases the risk for gastric cancer [93]. Similarly, Plummer and co-workers in large epidemiologic study showed a strong relationship between the presence of *H. pylori* DNA in gastric biopsies and the severity of precancerous lesions that is specific to CagA-positive strains [94]. Considering the role of virulence factors involved in the development of gastric cancer development: transported via T4SS system, released upon lysis in the gastric milieu or transported by OMV, apart from their expression, the geographical variability, diet and host-dependent factors should be included [95].

Besides CagA, VacA and OipA proteins, *H. pylori* OMVs contain different enzymes such as high temperature requirement (HtrA) serine protease, which induces E-cadherin cleavage resulting in the invasion of the gastric epithelium by *H. pylori*, and gamma-glutamyl transpeptidase (GGT), which participates in increasing gastric pH and glutamine synthesis [96, 97]. The production of some virulence factors associated with OMVs is upregulated in gastric mucosa due to exposure of *H. pylori* to a high concentration of reactive oxygen intermediates [18].

### Interaction of *H. pylori* OMVs with gastric epithelial cells

*H. pylori* OMVs were found under electron microscope in contact with the plasma membrane of human gastric mucosa cells as well as inside their endosomes, after the incubation with *H. pylori* broth culture filtrates [98, 99]. The uptake of *H. pylori* OMVs by gastric epithelial cells can occur via clatrin-dependent endocytosis or clatrin independent (lipid raft mediated mechanism), and strongly depends on adhesins exposed on OMVs. It was shown that the presence of VacA toxin may facilitate the OMVs uptake by host cells [99, 100]. Once OMVs bind to gastric epithelial cells, their components undergo endocytosis and are transported in endosomes, which undergo fusion with lysosomes, further impairing cell functions [99]. Ricci and co-workers showed that approximately 75% of VacA was released by *H. pylori* in a free-soluble form, while the remaining 25% was OMV-associated and that vacuolization in cell cultures was due to free-soluble VacA activity of a *H. pylori* broth culture filtrate [101]. This observation suggests that although OMV-associated VacA caused a statistically significant vacuolation, VacA encapsuled in OMVs could play a role different from that of free-soluble toxin such as an alternative delivery system to niches other than gastric mucosa (intestines), where the OMVs containing vesicles might interact with epithelial cells influencing their integrity. However, these aspects of alternative pathological role of OMVs caring VacA need to be further investigated in complex *in vitro* studies.

**Fig. 1:**
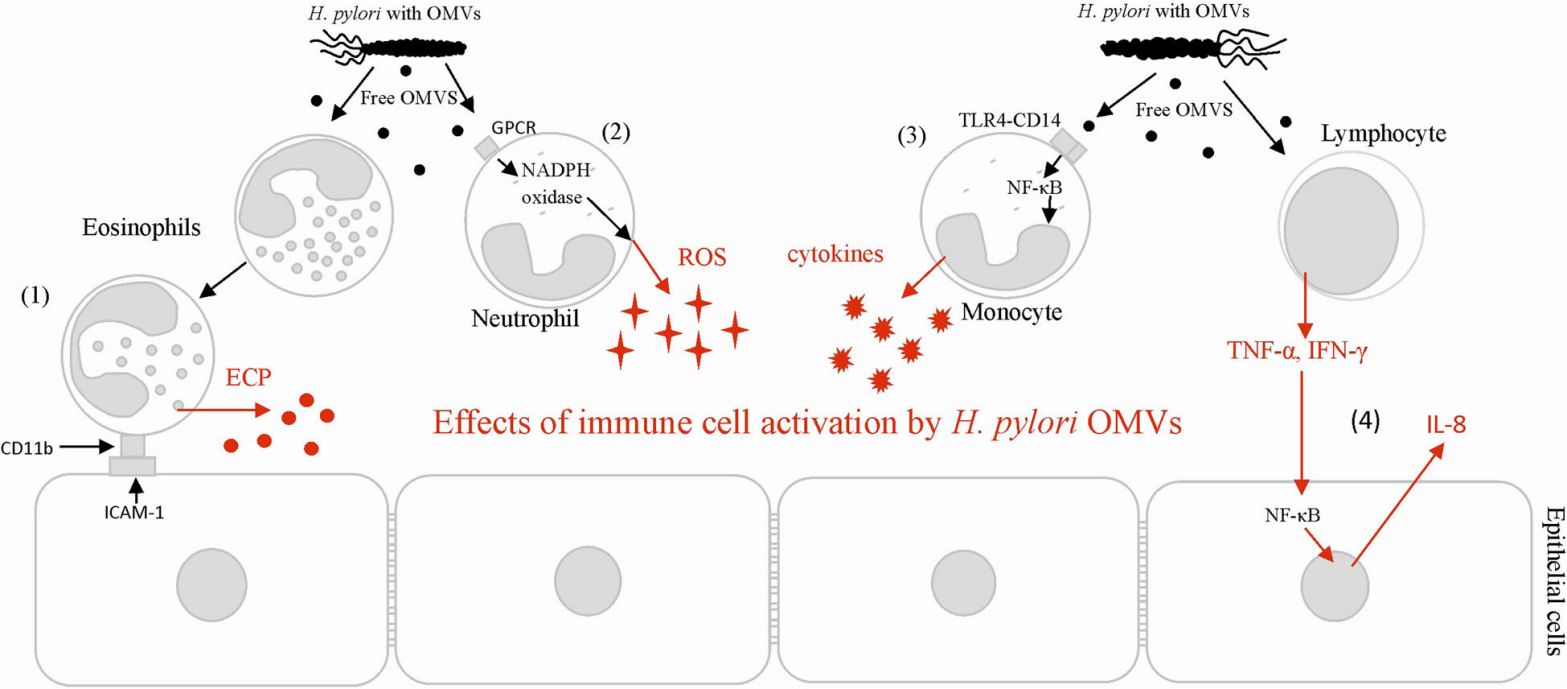
Various mechanisms of *H. pylori* outer membrane vesicles (OMVs)-mediated immune responses: (1) *H. pylori* OMVs induce ICAM-1 (intracellular adhesion molecule)-1 and CD11b (integrin) expression, causing degranulation and ECP (eosinophil cationic protein) release; (2) neutrophil activation via G-protein pathway involving GPCR (G-protein-coupled receptor) and kinases, resulting in oxidative burst and reactive oxygen species (ROS) production due to NADPH oxidase activation; (3) nuclear factor (NF)-κB activation and cytokine release by monocytes involve Toll-like receptor (TLR)4 and CD14 binding with *H. pylori* ligands delivered by OMV’S; (4) the increase in T-cell activation and cytokine secretion including TNF (tumor necrosis factor)-α and interferon (IFN)-γ during *H. pylori* infection stimulate the release of proinflammatory cytokines such as interleukin (IL)-8 from gastric epithelium through NFκB activation pathway

In response to vesicle-associated virulence factors, gastric and immune inflammatory cascade is induced (Fig. 1). Components of *H. pylori* OMVs can stimulate the production of both pro- and anti-inflammatory cytokines such as IL-6 and IL-10, respectively, and activate an oxidative burst [102, 103]. Nicotinamide adenine dinucleotide phosphate (NADPH) oxidase, which mediates this process, has been found to be activated in response to *H. pylori* OMVs components such as HP-NAP, which upregulates β2 integrin expression and attracts leukocytes to epithelial cells [104, 105]. *H. pylori* OMVs were also shown to induce human eosinophil degranulation and the release of eosinophil cationic protein (ECP), which enhance the expression of the intracellular adhesion molecule (ICAM)-1 on epithelial cells as well as CD11b integrin on eosinophils [102, 106]. *H. pylori* virulence factors delivered by OMVs can target transcription regulators such as a nuclear factor kappa-light-chain-enhancer of activated B cells (NF-κB), mitogen-activated protein kinase (MAPK) and extracellular signal-regulated kinase (ERK) of the host. On this way OMVs modulate the expression of genes, which products are involved in the inflammatory response, such as the secretion of IL-8, TNF-α or IL-1β or proliferation and carcinogenesis [107, 108]. It was shown that IL-8 production by gastric epithelial cells was stimulated by *H. pylori* OMVs independently of VacA, what suggests that vesicles without this exotoxin can cause cell damage due to induction of inflammatory response [109].

These examples show that *H. pylori* OMVs may strengthen the immune response which lead to diseases development or, on the contrary, promote downregulation of immune reactivity which support infection persistence. Still, the effects of *H. pylori* OMVs on the immune cells are poorly understood. Recently, the influence of *H. pylori* OMVs with dendritic cells (DC) was described. These interaction resulted in NF-κB dependent upregulation of heme oxigenase-1 (Ho-1), expression and potentially modulation of DCs function [110].

### *H. pylori* OMVs and disease development

*H. pylori* are non-invasive or low invasive and due to this secreted products of these bacteria or those introduced directly into the host cells play a role in the *H. pylori* induced diseases. *H. pylori* – related malignancy is strongly associated with the downregulation of host immunity and induction of structural and functional cell alterations that occur through *H. pylori* virulence factors [6, 111]. Numerous immune reactions do not require direct host-pathogen contact, thus OMVs of *H. pylori* may amplify its virulence. OMVs of *H. pylori* carrying the CagA affect cell-junctions and associated regulatory proteins, leading to chromatin changes, inappropriate transcription, and promotion of cancer development [112]. *H. pylori* OMVs containing CagA increases the affinity of ATP to H1 histone, which alters the binding of DNA and histones within the nucleosome [113]. Using transgenic mice expressing the *cagA* gene, treated with a colitis inducer-dextran sulfate sodium (DSS), it was shown that OMVs carrying CagA and VacA can promote inflammation [107]. Macrophages producing IL-1β and IL-8, as well as gastric epithelial cells secreting IL-8 were involved in that process. These proteins can also induce the expression of interferon (IFN)-γ and IL-17 [114, 115]. Moreover, by exosomes, which are eukaryotic cell-derived vesicles present in body fluids, CagA potentially may promote the development of extragastric diseases, including cardiovascular and haematologic diseases, parkinsonism and other disorders. It was shown that the addition of purified exosomes containing CagA to gastric epithelial cells induced cell elongation, implying that exosomes are functional CagA carriers [116]. Due to this proinflammatory potential of *H. pylori* OMVs, which were detected in gastric juice of patients with gastric cancer is suspected to participate in the process of carcinogenesis [114, 115]. Recently, Choi and co-workers evaluated the clinical significance of *H. pylori* OMVs on the pathogenesis of gastric malignancy. *H. pylori* OMVs from gastric juice samples obtained from healthy controls (HC) and patients with duodenal ulcer (DU), gastric ulcer (GU) or gastric cancer (GC) patients were analysed in the context of gastric cancer incidence. The analysis showed that both *H. pylori* cells and *H. pylori*-derived OMVs were present at a significantly higher level in the gastric juices of gastric cancer patients than in normal controls and that the *H. pylori*-derived OMVs induce inflammation in mice model, which is associated with gastric cancer development [114]. Transmission electron microscopy images showed that the OMVs were spherical in shape regardless of the disease type, however the authors did not present the differences in *H. pylori* OMV isolated from patients with different gastric outcomes. These results showed the association between OMVs presence and gastric lesions which should be further explored in more appropriate animal model (guinea pig, macaque). Chitcholtan and colleagues observed increased micronuclei formation, alteration in iron metabolism and gluthatione loss in AGS human gastric epithelial cells treated with *H. pylori* OMV, however this effect was observed only in OMV isolated from a toxigenic *H. pylori* strain [115].

*H. pylori* OMVs are rich in membrane proteins, porins, adhesins and several molecules known to modulate chemokine secretion, cell proliferation and other host cellular processes and constitute a vehicles for the CagA and VacA [117]. Furthermore, a recent analysis of the OMV proteome of 15 clinical isolates of *H. pylori* demonstrated that not all strains express the same proteins or at least not all the proteins were detected in the analysis of the different strains. Several membrane proteins, including Omp11 and BabA, were found to be expressed by all strains. Omp26 was expressed by all disease-related strains but was only present in one out of five strains from asymptomatic carriers, which makes Omp26 a potential target for further investigation in the search for proteins unique to disease-related *H. pylori* strains. The authors suggest that the presence of Omp30 and absence of Omp6 within *H. pylori* OMVs seemed to be associated with *H. pylori* strains causing duodenal ulcer [118]. It was also recently shown that the size of *H. pylori* OMVs determines its content. The proteomic analysis revealed that larger OMVs contain significantly more adhesins (Hop, SabA, BabA) compared to smaller OMVs, and that most of the *H. pylori* proteins related with metabolism are associated within smaller OMVs. Interestingly, proteins associated with *H. pylori* survival or virulence were common to both small and large OMVs, including: urease A and B subunits, neutrophil activating protein, VacA, and the porin HopA [119].

**Fig. 2:**
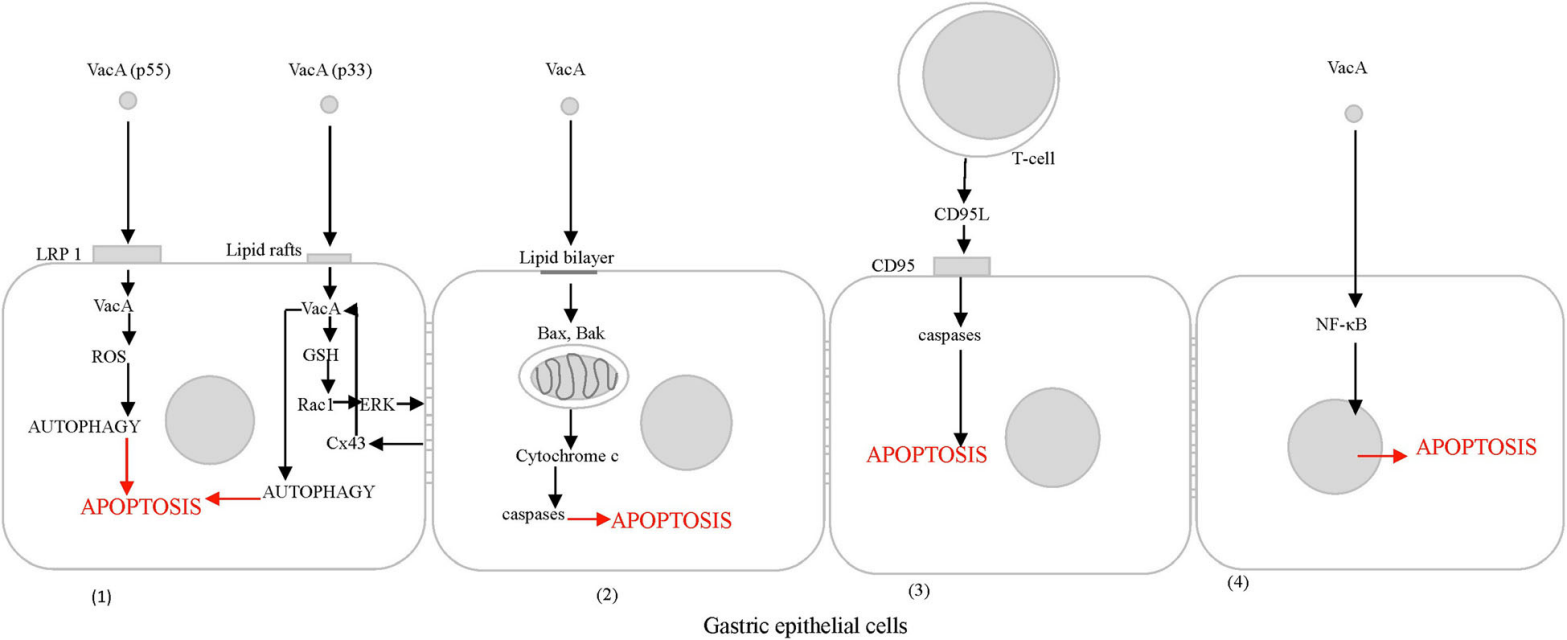
Distinct mechanisms of cell apoptosis induction by *H. pylori* VacA: (1) induction of apoptosis via autophagy, (2) cytochrome c release from mitochondria, (3) CD95-CD95L binding or (4) NFκB activation

**Fig. 3:**
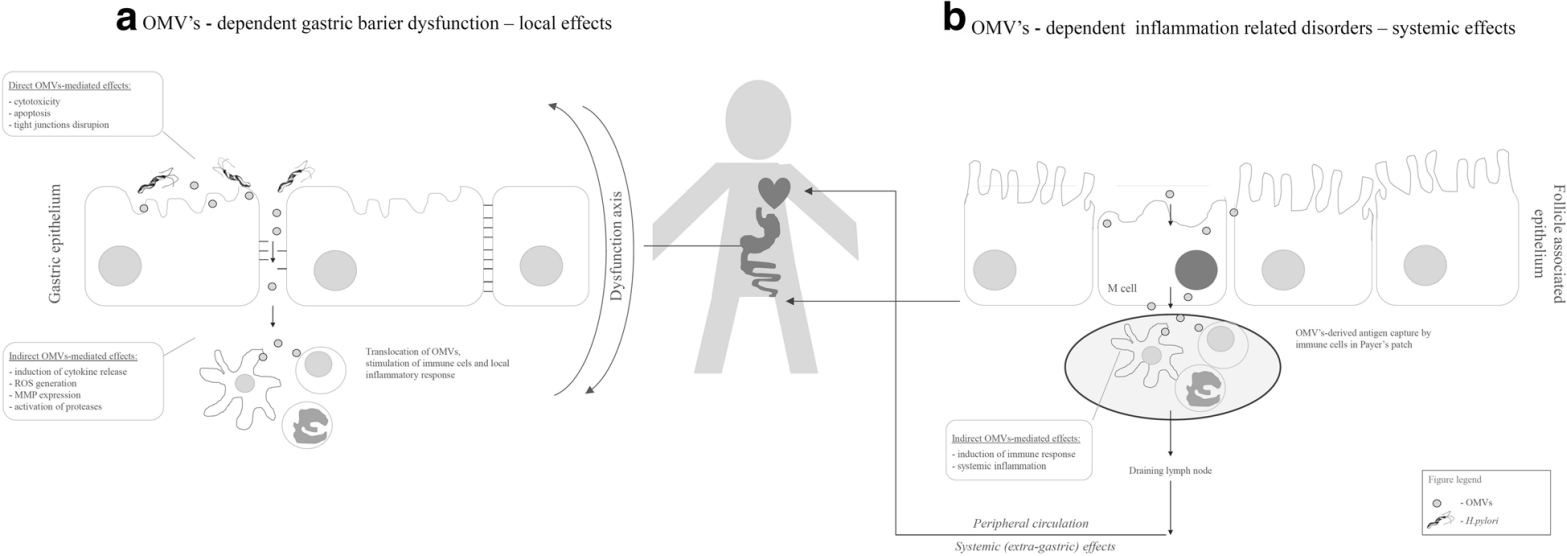
Local (**a**) and systemic (**b**) effects induced by *Helicobacter pylori* (*H. pylori*) outer membrane vesicles (OMVs). *H. pylori* OMVs induce gastric barrier dysfunction due to direct cytotoxicity, apoptosis or tight junctions disruption. Moreover, gastric barrier dysfunction axis may be deepen indirectly by the local inflammation due to accumulation of cytokine, reactive oxygen species (ROS), metaloproteinases (MMP) and other proteolytic enzymes. *H. pylori* OMVs also induce systemic effects, which may originate from capture of OMVs antigens by M cells, their presentation to immune cells in Payer’s patches and translocation into the circulation, where together with OMV’s-induced cytokines, indirectly induce systemic effects

As mentioned above, VacA, present in OMVs may bind to the lipid bilayer in the cell membrane of the host cells and form anion-selective and voltage-dependent channels that increase the efflux of complex molecules – this, in consequence may facilitate the growth of *H. pylori* [81, 120]. Soluble and OMVs bound VacA induces apoptosis in gastric epithelial cells via the mitochondria-dependent pathway, which is initiated by the cytochrome c release. This mechanism occurs indirectly due to VacA-mediated activation of proapoptopic Bcl-2 proteins: Bcl-2-associated X protein (Bax) and Bcl-2 homologous antagonist killer (Bak), which increase the permeability of the mitochondrial membrane [121]. Binding of the p55–58 VacA domain to host receptors, such as low density lipoprotein receptor-related protein 1 (LRP1) may also result in generation of reactive oxygen species (ROS) and the induction of cell autophagy followed by formation of autophagosome and then apoptosis [122]. Furthermore, the p33–37 VacA domain also induces apoptosis by the accumulation of connexin 43 (Cx43) in autophagic vesicles via the Ras-related C3 botulinum toxin substrate 1 (Rac1)/ERK dependent pathway, and similarly to CagA, VacA may induce NF-κB-dependent apoptosis [123, 124]. The induction of cell apoptosis in response to soluble or OMVs bound VacA is showed in Fig. 2. It has been found that supernatants from the broth cultures of *H. pylori*, mostly of toxigenic strains, induce apoptosis by the receptor-ligand pathway, which involves the CD95 ligand (CD95L) and the CD95 receptor (Fas) [125]. T-cells and the cytokines they release such as TNF-α and IFN-γ occur at higher levels in the gastric epithelium of *H. pylori*-infected patients, and increase local Fas expression [126]. *H. pylori* may also induce apoptosis via the interaction of soluble or OMVs bound urease to class II major histocompatibility complex (MHC II) molecules, which are expressed in epithelial cells and play a key role in pathogen recognition [127]. The ability of *H. pylori* components associated with OMVs to induce cell apoptosis may potentially contribute to the development of *H. pylori*-related diseases. Enhanced loss of epithelial cells due to *H. pylori*-induced apoptosis promote the development of gastric atrophy, which predisposes to neoplasia (Fig. 3a). Alternatively, apoptosis may be a response to hyperproliferation accompanied by an increased prevalence of mutations – as a way to reduce excessive growth of abnormal cells. It should be taken into account that apoptosis may also protect against deleterious effects of inflammatory response [128]. On the other hand, the induction of apoptosis in lymphocytes or antigen presenting cells by *H. pylori* components, free or OMVs bound, may diminish clonal expansion and the effectiveness of specific anti-*H. pylori* immune responses [129, 130].

The *H. pylori* components present in OMVs such as LPS may induce opposite effects. The endotoxic activity of *H. pylori* LPS is lower than the activity of LPS of other Gram-negative bacteria due to distinct structure of lipid A. Thus OMVs decorated with *H. pylori* LPS may downregulate the inflammatory response or lead to it’s upregulation by the induction of fibrinolytic components [131, 132]. *H. pylori* LPS due to presence of Lewis antigens in O-specific polysaccharide chains, which share a common structure with blood group-related components of the host may lead to generation of autoimmunity due to the stimulation of autoantibody production [103]. Lewis antigens present in LPS bound to OMVs can contribute to chronic stimulation of the host immune cells. The ability of OMVs to bind and diminish the level of anti-Lewis antibodies from the sera of *H. pylori* infected individuals indicate that OMVs may potentially play a role in autoimmune processes related to *H. pylori* infection [69]. The above examples show that *H. pylori* OMVs may strengthen the immune response leading to diseases development or promote reduction of immune reactivity and thus support the infection persistence.

*H. pylori* OMVs due to the induction of gastric barrier instability and dysbiosis can be translocated to deeper layers of gastric mucosa and gastrointestinal tract. OMVs have a proper size (20–200 nm) to enable their entry by circulation into lymph vessels and uptake by antigen presenting cells. Smaller OMVs ranging from 20 to 100 nm in size preferentially entered host cells *via* caveolin-mediated endocytosis. Whereas larger OMVs ranging between 90 and 450 nm in size entered host epithelial cells *via* macropinocytosis and endocytosis [133]. Several biological functions have been suggested for natural OMVs. Naturally occurring OMVs may function as long distance delivery system of specific components (Fig. 3b) [134]. This natural feature of bacterial OMVs is already used in medicine to deliver vaccine components to different sites in the organism, including circulation e.g. OMVs are used in the first generation vaccine product that has been approved for the market as well as for drug-delivery development including anti-*H.pylori* therapy studied on animal model [135]. Considering that, one can hypothesize that OMVs present in the circulation, by the interaction with epithelial cells and leukocytes, might affect the responsiveness of other immune cells of the host [136]. One class of vesicles termed exosomes is produced by exocythosis of multivesicular bodies. It was showed that CagA is present in exosomes derived from serum of patients infected with *cagA*-positive strains [137]. These may suggest that secreted exosomes may enter a bloodstream, and be delivered to different organs. Other group reported that *H. pylori* OMV localizes in a close vicinity of the tight junction protein ZO-1 and induce changes connected with cell barrier instability [138]. This way of translocation of *H. pylori* virulence factors can elucidate the involvement of *H. pylori* compounds in the development of local as well as systemic inflammation - crucial for the initiation or the maintenance of gastric and extragastric inflammatory diseases [6, 15, 139, 140].

### Conclusions and future research

In conclusion, outer membrane vesicles are continuously shed by *H. pylori* from the surface of these bacteria during infection. The composition of *H. pylori* OMVs and their ability to translocate via gastric barrier to intestines and into circulation suggests that they may play a central role in *H. pylori* pathogenesis by acting as delivery vehicles to transport virulence factors. Locally, OMVs induce direct effects such as tight junction disruption, cytotoxicity or apoptosis or indirectly affect the immune cells to release cytokines, proteases or tissue damaging factors such as MMP or ROS. Their translocation from gastric niche through digestive system into intestines may possibly lead to a capture and presentation by M cells to other immune cells and result with the immunomodulation of immune mechanisms leading to spread of OMVs into circulation. The studies concerning the involvement of OMVs produced by *H. pylori* in the pathogenesis of extra-gastric diseases such as coronary heart disease or food allergies might explain their importance and mechanisms by which they promote inflammation or tolerance.

## Abbreviations

ABC: ATP-binding cassette
Alp: Adherence associated lipoproteins
BabA: Blood group antigen-binding adhesin A
CagA: Cytotoxin-associated gene A
CD95L: CD95 ligand
Cx43: Connexin 43
DC: Dendritic cell
DSS: Dextran sulfate sodium
ECP: Eosinophil cationic protein
eDNA: Extracellular DNA
ERK: Extracellular signal-regulated kinase
ETEC: Enterotoxigenic *E. coli*
GGT: Gamma-glutamyl transpeptidase
GPCR: G-protein-coupled receptor
Ho-1: oxygenase-1
HP-NAP: *H. pylori* neutrophil activating protein
HtrA: High temperature requirement A
ICAM-1: Intracellular adhesion molecule-1
IFN: Interferon
IL: Interleukin
IM: Inner membrane
Le: Lewis antigenic determinant
LPC: Lysophosphatidylcholine
LPE: Lysophosphatidyl ethanolamine
LPS: Lipopolysaccharide
LRP1: Lipoprotein receptor-related protein 1
MAPK: Mitogen-activated protein kinase
MHC: Major histocompatibility complex
MMP: Metalloproteinase
NADPH: Nicotinamide adenine dinucleotide phosphate
NFκB: Nuclear factor kappa-light-chain-enhancer of activated B cells
OipA: Outer inflammatory protein A
OM: Outer membrane
OMPs: Outer membrane proteins
OMVs: Outer membrane vesicles
PC: Phosphatidylcholine
PE: Phosphatidylethanolamine
PG: Phosphatidylglycerol
pOMVS: Planktonic OMVs
Rac1: Ras-related C3 botulinum toxin substrate 1
ROS: Reactive oxygen species
SabA: Sialic acid-binding adhesin
T4SS: Type IV secretion system
TLR: Toll-like receptor
TNF: Tumor necrosis factor
VacA: Vacuolating cytotoxin A
